# The regulatory role of APE1 in epithelial‐to‐mesenchymal transition and in determining EGFR‐TKI responsiveness in non‐small‐cell lung cancer

**DOI:** 10.1002/cam4.1717

**Published:** 2018-08-14

**Authors:** Xiao Yang, Yu Peng, Xuan Jiang, Xianfeng Lu, Wei Duan, Shiheng Zhang, Nan Dai, Jinlu Shan, Yan Feng, Xuemei Li, Yi Cheng, Yuxin Yang, Laura Baugh, Gianluca Tell, Dong Wang, Mengxia Li

**Affiliations:** ^1^ Cancer Centre Daping Hospital and Research Institute of Surgery Third Military Medical University Chongqing China; ^2^ Department of Pathology Baylor University Medical Center Dallas Texas; ^3^ Laboratory of Molecular Biology and DNA Repair Department of Medical and Biological Sciences University of Udine Udine Italy

**Keywords:** apurinic/apyrimidinic endonuclease 1, EGFR‐TKI resistance, epithelial‐to‐mesenchymal transition, non‐small‐cell lung cancer, transforming growth factor‐β

## Abstract

**Background:**

Epithelial‐to‐mesenchymal transition (EMT) plays a pivotal role in resistance to EGFR tyrosine kinase inhibitors (TKIs) in non‐small‐cell lung cancer (NSCLC). Our previous study revealed that in osteosarcoma, human apurinic/apyrimidinic endonuclease 1 (APE1) regulates transforming growth factor‐β (TGF‐β), an important player in EMT. We therefore hypothesized a link between APE1 and EGFR‐TKI responsiveness in NSCLC.

**Methods:**

The protein levels of APE1 were analyzed in tumors of NSCLC patients receiving EGFR‐TKI treatment. The correlation between APE1 expression and progression‐free survival (PFS), overall survival (OS), or response rate were analyzed. The impact of APE1 on the response to EGFR‐TKIs was measured by exogenous manipulation of APE1 in EGFR‐TKI‐sensitive and EGFR‐TKI‐resistant cells.

**Results:**

We indicate that low expression of APE1 in tumors is associated with a significantly longer PFS (20.8 months vs 8.4 months, *P *=* *0.008) and a preferential OS (39.0 months vs 17.0 months, *P *=* *0.001), with no difference in initial response rate to EGFR‐TKIs. We observed that APE1 protein level was significantly increased in EGFR‐TKI‐resistant cells and was associated with downregulated E‐cadherin and upregulated vimentin. The EMT phenotype, as well as the levels of TGF‐β, was suppressed in APE1 knockdown HCC827/IR and PC‐9/ER cells, while the EMT phenotype was promoted in APE1‐overexpressed HCC827 and PC‐9 cells. Furthermore, a specific APE1 redox inhibitor (ie, E3330) effectively reversed the EMT phenotype and further sensitized the cells to EGFR‐TKIs.

**Conclusion:**

This study revealed a significant role of APE1 in EGFR‐TKI resistance via novel regulatory effects on the EMT phenotype in NSCLC.

## 1 INTRODUCTION

The introduction of EGFR tyrosine kinase inhibitors (EGFR‐TKIs), including gefitinib, erlotinib, and icotinib,^1^ has successfully improved the response rate, progression‐free survival, and overall survival of advanced non‐small‐cell lung cancer (NSCLC) patients from <1 year to up to 3.5 years in patients carrying driver gene mutations. Oral administration of EGFR‐TKIs is now a standard first‐line treatment for NSCLC patients with activating EGFR mutations, such as exon 19 deletions and the L858R point mutation. Unfortunately, patients with a promising initial response rate (~75%) usually develop acquired resistance to EGFR‐TKIs within 8‐10 months, which limits continuous clinical benefit. Optimal treatment after progression is not clearly defined. Collective efforts have been made to understand the mechanisms of resistance to EGFR‐TKIs in order to overcome or, at least, attenuate the development of acquired resistance. Although secondary mutations, such as the T790M gatekeeper mutation in the EGFR tyrosine kinase domain, account for half of the resistance to first‐generation EGFR‐TKIs, the remaining mechanisms and key players are not yet understood.

During the process of acquired EGFR‐TKI resistance of NSCLC, the epithelial‐to‐mesenchymal transition (EMT) program is considered to be activated, as shown in recent clinical studies.^2^, ^3^, ^4^, ^5^ For instance, Sequist et al^3^ analyzed biopsy samples from 37 cases of drug‐resistant NSCLC patients carrying EGFR mutations, and a pronounced EMT was observed in a subset of EGFR‐TKI‐resistant specimens. In agreement with these findings in clinical specimens, NSCLC cell lines with activating EGFR mutations may develop an EMT phenotype upon long‐term exposure to erlotinib or gefitinib.^6^, ^7^ EMT is a cellular program characterized by loss of an epithelial gene expression signature, such as E‐cadherin, and gain of activation of genes that partially define mesenchymal features, such as vimentin. EMT is observed in normal development and associated with enhanced invasiveness, metastatic behavior, and EGFR‐TKI resistance of epithelial cancers. The importance of EMT in EGFR‐TKI resistance is highlighted by the observations of Chung et al^4^ that EMT developed in a lung cancer patient who had an acquired resistance to erlotinib, while there were no known resistance mechanisms such as a T790M mutation or MET amplification. This observation suggests that the activation of EMT could independently trigger EGFR‐TKI resistance. However, the detailed regulatory mechanisms of EMT are just beginning to be understood.

Alterations of the gene expression profile during EMT, as well as nontranscriptional changes, are regulated by the convergence of signaling pathways that respond to extracellular stimuli. Among these, transforming growth factor‐β (TGFβ) signaling plays a predominant role in inducing EMT. In carcinomas, activation of the TGFβ1 signaling pathway promotes an epithelial plasticity response, which further triggers EMT. It is now known that TGFβ1 promotes EMT through Smad‐dependent transcriptional regulation of HMGA2, which further induces expression of the important EMT‐associated transcription factors Snail, Slug, and Twist.^8^ Our previous report showed that human apurinic/apyrimidinic endonuclease 1 (APE1) promotes transcriptional activation of TGFβ1 expression in osteosarcoma cells.^9^ We have clearly shown that APE1 knockdown downregulated TGFβ1 mRNA, intracellular protein, and secretion levels, which further attenuated in vitro angiogenesis. As osteosarcoma is a mesenchymal‐derived malignancy, we were unable to observe the epithelial plasticity or EMT phenotype, but we hypothesized that APE1 could contribute to regulation of EMT in epithelial cancers. However, to our knowledge, there is no direct evidence demonstrating the association of APE1 with EMT.

APE1 is an essential enzyme in DNA base excision repair (BER), which primarily repairs oxidative lesions and alkylation of bases as well as spontaneous hydrolysis of bases. More importantly, the APE1 protein contains reduction‐oxidation (redox)‐sensitive cysteine residues, which respond to redox alterations in the microenvironment, which in turn alters the DNA binding activities of transcription factors, including AP‐1, NF‐κB, p53, HIF‐1α, and NRF‐1.^10^ Considering the downstream target genes that these transcription factors regulate, APE1 plays a fundamental role in the cellular response to oxidative stress and other redox‐related stimuli. There is an increasing body of evidence showing that EMT is also a redox‐responsive process and that the critical transcription factor Snail is regulated by ROS production (reviewed in 11). Thus, it is reasonable to hypothesize that APE1 may participate in an EMT process, based on the redox responsiveness of EMT and our previous experimental data on TGFβ1 regulation. Therefore, in our current study, we investigated the relationship existing between APE1 expression and EGFR‐TKI acquired resistance mediated by EMT activation in NSCLC biopsy tissue. In addition, we also analyzed this relationship in established EGFR‐TKI‐resistant cell lines. The study was designed to elucidate how APE1 affects the expression of specific epithelial and mesenchymal tissue markers in the EMT process and which function of APE1 is responsible for this effect. Furthermore, we tested whether the APE1 inhibitors may restore responsiveness to EGFR‐TKIs in the acquired resistant cell lines via inhibition of EMT. This study, to the best of our knowledge, shows the first evidence confirming the role of APE1 in EGFR‐TKI resistance via promoting EMT and further highlights the therapeutic potential of APE1 inhibitors to decrease EGFR‐TKI resistance.

## 2 MATERIALS AND METHODS

### 2.1 Study subjects

The study group consisted of 101 advanced NSCLC patients (48 male and 53 female, mean age 59.5 years) either carrying an activating EGFR gene mutation or with unknown EGFR gene status but with potential benefit from EGFR‐TKIs.^12^ All patients were from the Chinese Han population and consecutively enrolled from January 2011 to December 2013 in Daping Hospital, Third Military Medical University (Chongqing, China), without restriction of age, gender, pathology, or stage. All patients received standard first‐line administration of gefitinib, erlotinib, and icotinib without any chemotherapy or localized treatment. At recruitment, informed consent was obtained from each patient, and each participant was then interviewed to solicit detailed information on demographic characteristics. This study was approved by the ethics committee of Daping Hospital. All patients were followed up for more than 36 months.

### 2.2 Cell culture and human tissue samples

Human lung adenocarcinoma cell line HCC827, carrying the EGFR gene exon 19 deletion, and its in vitro induced icotinib‐resistant derivate HCC827/IR were obtained from the R&D Department of Betta Pharmaceuticals Co., Ltd.^13^ Another human lung adenocarcinoma cell line PC‐9, carrying the EGFR gene exon 19 deletion, and its in vitro induced erlotinib‐resistant derivate PC‐9/ER were kind gifts from Dr. Bo Zhu from Xinqiao Hospital, Third Military Medical University. HCC827 and HCC827/IR cells were grown in RPMI‐1640 (HyClone Laboratories Inc., LOGAN, UT, USA) supplemented with 10% FBS, while PC‐9/ER, as well as their parental sensitive cells, were cultured in DMEM (HyClone) with 10% FBS.

### 2.3 Targeted genomic capture and next‐generation sequencing (NGS)

The NGS assays of HCC827/IR and PC‐9/ER cells were carried out in facility of MyGenostics biotech company (Beijing, China). Whole‐exon regions of the target genes were enriched using the MyGenostics Target Region Enrichment protocol. Three micrograms of genomic DNA were fragmented by nebulization and repaired. Illumina adapters were then ligated to the fragments, and the final size was 350‐400 bp. The enrichment libraries were sequenced on Illumina HiSeq 2000 sequencer for paired read 100 bp. Next, high‐quality reads were identified using SOAPaligner software, respectively. Variants were first selected using SOAPsnp software,^14^ and then, they were further processed according to the dbSNP (v130) database and CCDS. Subsequently, the reads were realigned to the reference genome (NCBI36.3) using BWA software.^15^ SNPs and indels were identified using GATK programs.

### 2.4 APE1 overexpression and knockdown

For exogenous overexpression of APE1, cultured cells were transfected with an APE1 eukaryotic expression vector, p3XFLAG‐CMV/APE1WT^16^ or control empty backbone vector using X‐tremeGENE 9 DNA Transfection Reagent (Roche Diagnostics, Shanghai, China) following the manufacturer's protocol. Cells were then cultured for another 72 hours before protein expression level determination or phenotype measurements. For APE1 knockdown experiments, cells were transfected with 0.2 nmol/L APE1‐siRNA; the sequences for APE1‐siRNA1: 5′‐UACUCCAGUCGUACCAGACCU‐3′, 5′‐GUCUGGUACGACUGGAGUACC‐3′, APE1‐ siRNA2: 5′‐CUCAAUGUGGCACAUGAAG‐3′, 5′‐CUUCAUGUGCCACAUUGAG‐3′, and for scramble‐siRNA: 5′‐GACCAUGCUGACCUCAUGGAA‐3′, 5′‐CCAUGAGGUCAGCAU GGUCUG‐3′. These siRNAs were transfected with Lipofectamine 2000 (Invitrogen, Carlsbad, CA, USA) according to the manufacturer's instructions. After 48 hours, the cells were harvested for protein expression level determination or phenotype measurements.

### 2.5 Cell viability assay

Cell viability was determined after drug treatments with/without APE1 manipulation using the cell counting kit‐8 (CCK‐8, Beyotime Inst. Biotech, China) according to the manufacturer's instructions.

### 2.6 Western blot analysis and antibodies

For whole‐cell extracts, cells were washed with ice‐cold phosphate‐buffered saline and collected by scraping. Cell pellets were homogenized in extraction buffer (50 mmol/L Tris‐HCl, 0.1% sodium dodecyl sulfate [SDS], 150 mmol/L NaCl, 100 μg/mL phenylmethylsulfonyl fluoride, 1 μg/mL aprotinin, 1% nonidet P‐40, and 0.5% sodium orthovanadate), then incubated at 4°C for 20 minutes, and centrifuged for 20 minutes at 12 000 *g*. The protein levels in the extracts were quantified using the Bio‐Rad DC protein assay. For Western blotting, whole‐cell extracts (20 μg/lane) were resolved on 8%‐12% SDS‐polyacrylamide gels and transferred onto nitrocellulose membrane (0.45 μm; Millipore, Bedford, MA, USA) in 25 mmol/L Tris‐base, 190 mmol/L glycine, and 20% methanol using a semidry blotter. Membranes were blocked with 8% fat‐free milk and 0.1% Tween 20 in Tris‐buffered saline. The APE1 monoclonal antibody was diluted 1:3000 while the E‐cadherin (Abcam, Shanghai, China) and vimentin (Cell Signaling Technology, Shanghai, China) monoclonal antibodies were used at the 1:1000 dilution recommended by the suppliers. The membranes were then incubated with a horseradish peroxidase‐conjugated secondary antibody (1:2000) (Pierce, Rockford, IL, USA). The proteins were detected using an enhanced chemiluminescence detection system (Pierce), and light emission was captured on X‐ray films (Kodak, Rochester, NY, USA).

### 2.7 Immunofluorescent staining

Cells growing on the chamber slides for attachment were subjected to various treatment before fixation using 4% paraformamide/PBS (w/v) at room temperature for 15 minutes. Cells were incubated with permeabilizing solution (0.1% Triton X‐100 in PBS, v/v) for 5 minutes. Cells were then blocked with 1% BSA at RT for 1 hour followed by overnight incubation with primary antibodies at 4°C in a humidity chamber. On the next day, the cells were washed with PBST for 15 minutes on a rocking board and then incubated with secondary antibodies (1:200 dilution) for 1 hour at room temperature. After washing with PBST, cells were incubated with DAPI for 5 minutes to stain the nuclei. Finally, cells were mounted with antifade mounting medium and images were captured using a LSCM laser scanning confocal microscope).

### 2.8 Enzyme‐linked immunosorbent assay (ELISA)

The 24‐hour cell‐free supernatant of tissue culture from PC‐9 with or without APE1 overexpression and PC‐9/ER with or without APE1 knockdown were collected 48 hours post‐transfection or infection and measured using a TGF‐β1 ELISA kit (Invitrogen) following the manufacturer's instruction. The absorbance of each well was read at 450 nm.

### 2.9 Electrophoretic mobility shift assay (EMSA)

EMSA was performed according to the LightShift chemiluminescence EMSA kit with minor modifications. Briefly, the nuclear extracts were incubated with 3′‐biotin‐labeled single‐stranded telomeric DNA probes. The probes containing the STAT3 consensus (STAT3F, 5′‐GATCCTTCTGGGAATTCCTAGATC‐3′, and STAT3R, 3′‐CTAGGAAGACCCTTAAGGATCTAG‐5′) or the HIF‐1α consensus (HIF‐1αF, 5′‐TCTGTACGTGACCACACTCACCTC‐3′, and HIF‐1αR, 3′‐AGACATGCACTGGTGTGA GTGGAG‐5′) were synthesized (Beyotime, Shanghai, China). After incubation, the samples were separated on a 5% polyacrylamide gel at 100 V for 60 minutes and then transferred to a Zeta‐Probe GT nylon membrane (Bio‐Rad). The probes were detected by HRP‐conjugated streptavidin and visualized by ECL reagents provided with the kit. The bands were quantified by Quantity One imaging software (Bio‐Rad, Hercules, CA, USA).

### 2.10 In vivo experiments

Male BALB/c nude mice between 4 and 5 weeks old were used for this study. Cell suspensions of HCC827/IR (1 × 10^6^) were inoculated subcutaneously to both groins of each nude mouse (20 mice in total). When the xenografts grew to approximately 250 mm^3^ on day 9 after cell inoculation, 18 tumor‐bearing mice were randomized into either the control group, icotinib group or E3330 + icotinib group (six mice per group). Icotinib (70 mg/kg) was injected abdominally once a day in the icotinib group, while E3330 (25 mg/kg) and icotinib (70 mg/kg) were injected abdominally once a day in the E3330 + icotinib group. After 12 days of continuous administration, the nude mice were sacrificed, and histologically, intact tumor tissues were collected. Tumor sizes were measured before each treatment and after the mice were sacrificed and calculated according to the formula: tumor size (mm^3^) = (maximum diameter ×minimum diameter^2^)/2.

### 2.11 Immunohistochemistry

Sections from paraffin‐embedded tumors were incubated with the indicated EMT marker antibodies overnight at 4°C and then rinsed with PBS and incubated with secondary antibody. Sections were rinsed with PBS and developed with diaminobenzidine substrate, and then counterstained with diluted Harris hematoxylin. Positive staining was detected as a brown color of the cells. A random 10 high power fields or at least 1000 tumor cells were counted and graded as follows: negative (−): positive cells rate <10%; positive (+): positive cells rate ≥10%; positive (++): positive cells rate ≥30%; positive (+++): positive cells rate ≥60%. Negative (−) and positive (+) were defined as low expression, while positive (++) and (+++) were defined as high expression.

### 2.12 Statistical analysis

All continuous data were expressed as the mean ± SD. Statistical analysis of numerical variables was assessed using the Student's *t* test and one‐way ANOVA, while the categorical variables were performed with chi‐square test. The univariate survival analysis was conducted by log‐rank test, and the multivariate one was conducted by COX hazard regression analysis. All statistical performance was achieved through SPSS 13.0. Differences with *P *<* *0.05 were considered as statistically significant.

## 3 RESULTS

### 3.1 Overexpression of APE1 is associated with EGFR‐TKI resistance in NSCLC patients carrying activating EGFR mutants

**Figure 1:**
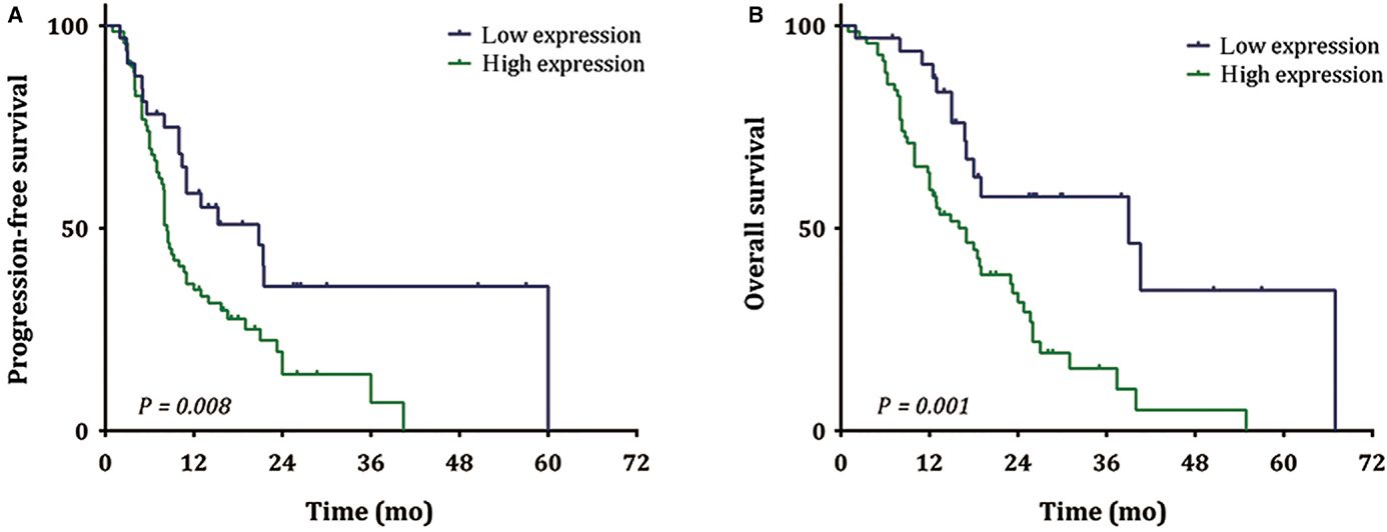
Association of APE1 protein level in NSCLC tissue and PFS and OS of patients receiving EGFR‐TKI. The expression of APE1 protein from 101 advanced NSCLC patients either carrying an activating EGFR gene mutation or with unknown EGFR gene status but with potential benefit from EGFR‐TKI was determined in biopsy tissue by IHC assay. The PFS (A) and OS (B) comparisons between different expression levels of APE1 were indicated in the Kaplan‐Meier survival curves, and the *P* value of each comparison was shown at the left corner

To correlate APE1 protein levels in the tumors of NSCLC patients with their responses to first‐generation EGFR‐TKIs, the expression of APE1 protein was analyzed in biopsy tissue by IHC. The analysis was performed on a cohort of 101 advanced NSCLC patients either carrying an activating EGFR gene mutation or with unknown EGFR gene status but with potential benefit from EGFR‐TKIs.^12^ All patients received standard first‐line administration of gefitinib, erlotinib, and icotinib without any chemotherapy or localized treatment. The clinicopathological characteristics are summarized in Table 1. Kaplan‐Meier survival curves show that low‐expression of APE1 is associated with an attenuated resistance represented by a significantly longer PFS (20.8 months vs 8.4 months, *P* = 0.008), while eventually reflecting a benefit on the overall survival (OS) (39.0 months vs 17.0 months, *P* = 0.001) (Figure 1). We also evaluate the association between APE1 expression and survival through COX hazard regression model (likelihood ration forward test), which shows that APE1 could be an independent negative factor for both PFS and OS (Table 2). The patients with high APE1 expression have a shorter PFS and OS, compared with those with low APE1 expression. These results imply that APE1 might play an important role in initiating or promoting resistance to EGFR‐TKIs in NSCLC, while its mechanism remains unclear.

**Table 1:** Clinicopathological characteristics of patients

Characteristics	Number of patients	Percentage
Age		
Average ± SD	59.48 ± 11.82	
<60	56	55.45
≥60	45	44.55
Gender		
Male	48	47.52
Female	53	52.48
Pathology		
Squamous cell carcinoma	5	4.95
Adenocarcinoma	88	87.13
Other	8	7.92
Stage^a^		
I+II	1	1.39
III+IV	71	98.61
Drug		
Gefitinib	44	43.56
Erlotinib	51	50.50
Icotinib	6	5.94
Response		
CR	2	1.98
PR	54	53.47
SD	31	30.69
PD	14	13.86
Smoking status^b^		
Smoker	75	76.53
Nonsmoker	23	23.47
EGFR mutation^c^		
Positive	67	94.37
Negative	4	5.63
APE1 expression		
High expression	72	71.29
Low expression	29	28.71

a Twenty‐nine subjects were unable to evaluate exact stage.

b Two subjects were unable to confirm whether they ever smoked.

c Thirty subjects were unable to evaluate the mutation condition of EGFR.

**Table 2:** Multivariant analysis for PFS and OS of patients

Characteristics	PFS		OS	
	HR (95%CI)	*P* value	HR (95%CI)	*P* value
Age		0.429		0.841
Gender		0.254		0.855
Pathology	2.090(1.068‐4.089)	0.031^d^	2.588(1.275‐5.253)	0.008^d^
Stage^a^		0.614		0.722
Drug		0.449	0.461(0.240‐0.888)	0.020^d^
Smoking status^b^		0.334		0.700
EGFR mutation^c^		0.946		0.487
APE1 expression	2.998(1.229‐7.314)	0.016^d^	4.724(1.564‐14.267)	0.006^d^

a Twenty‐nine subjects were unable to evaluate exact stage.

b Two subjects were unable to confirm whether they ever smoked.

c Thirty subjects were unable to evaluate the mutation condition of EGFR.

d *P *<* *0.05

### 3.2 APE1 level is elevated in EGFR‐TKI‐resistant cell lines and regulates cellular responses to EGFR‐TKIs

**Figure 2:**
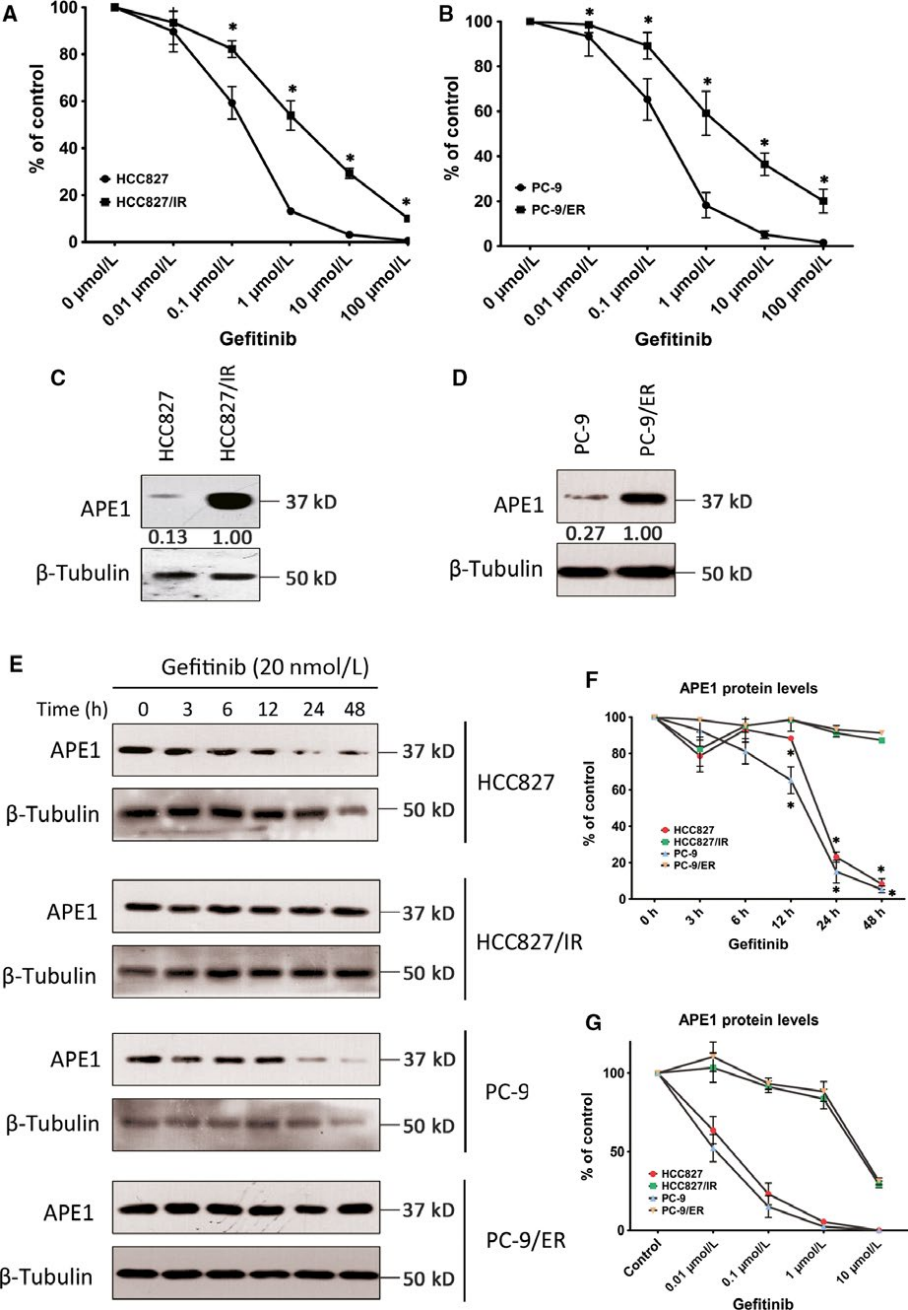
APE1 level elevated in EGFR‐TKI‐resistant cell lines. Two established acquired resistant cell lines, HCC827/IR and PC‐9/ER, as well as their parental EGFR‐TKI responsive cells, were subjected to CCK8 assay to determine the responsiveness of different cells to increasing concentrations of gefitinib (A and B), * indicates statistically significant difference when compared with the same treatment dose of parental cell (*P *<* *0.01). HCC827/IR and PC‐9/ER cells, as well as their parental EGFR‐TKI‐responsive cells, were treated with 20 nmol/L gefitinib (representative blots shown in C and D) for 48 h or with increasing concentrations of gefitinib (representative blots shown in E), harvested, and analyzed by Western blot for APE1 protein levels. APE1 expression levels were assayed by Western blot in EGFR‐TKI‐resistant HCC827/IR and PC‐9/ER, as well as their parental cells (F and G, respectively), * indicates a statistically significant difference when compared with the DMSO treated cells (*P *<* *0.01). The mean values of at least three individual repeated experiments are shown as the mean ± SD

**Figure 3:**
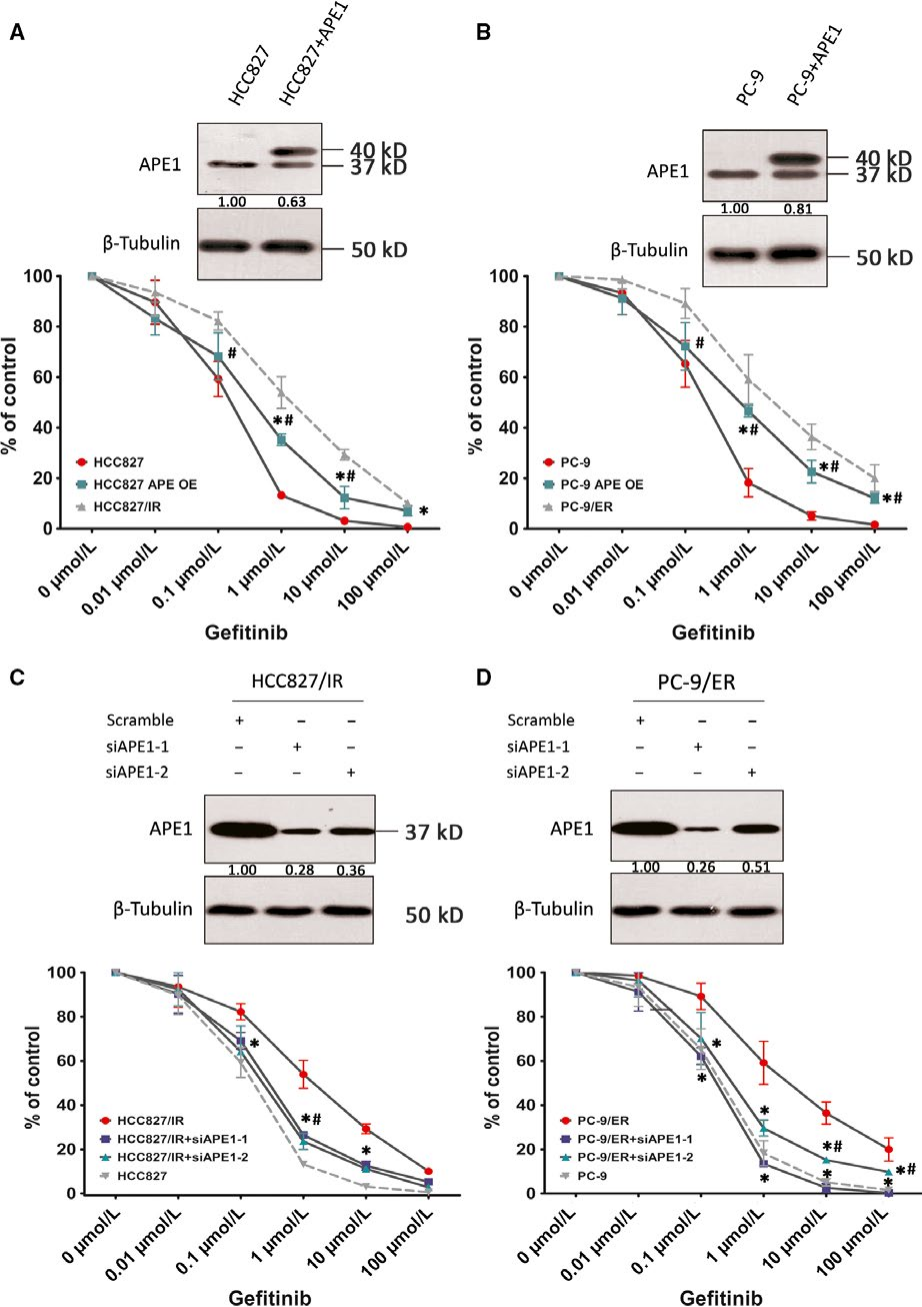
Manipulations of APE1 regulate cellular responses to EGFR‐TKIs. APE1 was overexpressed in HCC827 and PC‐9 cell lines via lentiviral particles (A and B) and knocked down in HCC827/IR and PC‐9/ER via two different siRNA sequences specific for the Ape1 gene (C and D). APE1 expression levels were then analyzed by Western blot (shown in the upper panel of each subfigure) and treated with increasing concentrations of gefitinib to determine the cytotoxicity of EGFR‐TKI by CCK8 assay. To exclude the impact of APE1 manipulation on cell growth, various gefitinib dose treatments of each group have been normalized to the readout of 0 uM (DMSO only) treatment. Mean values of at least three individual experimental repeats are shown as the mean ± SD. In A and B, statistically significant differences of the APE1 overexpression group when compared with empty particle‐infected parental sensitive cells (indicated by *) or resistant cells (indicated by #) at the same treatment dose/time (*P *<* *0.01) are shown. In C and D, statistically significant differences of APE1 knockdown group when compared with empty particle‐infected resistant cells (indicated by *) or parental sensitive cells (indicated by #) at the same treatment dose/time are shown (*P *<* *0.01)

To explore the role of APE1 in the cellular response to EGFR‐TKI, APE1 protein levels following EGFR‐TKI treatment were initially determined in NSCLC cells. To distinguish the different responses in EGFR‐TKI‐sensitive and EGFR‐TKI‐resistant cells, two established, acquired resistant cell lines, HCC827/IR and PC‐9/ER, as well as their parental sensitive cells were utilized (the resistant features are examined by CCK‐8 and shown in Figure 2A,B). We detected no T790M mutation, MET amplification or other known resistant‐related gene alteration in both TKI‐resistant cell lines by NGS. As shown in Figure 2C,D, basal APE1 protein levels are significantly increased to more than 10‐fold in both resistant cell lines when compared to their parental cells. Though APE1 downregulated in response to EGFR‐TKI in sensitive cells at 48 hours probably is due to cell death, we can still see a veritable response to EGFR‐TKIs at both 12 and 24 hours (*P* < 0.01), but not in resistant cells, suggesting that the compromised APE1 expression facilitates the cytotoxicity of EGFR‐TKIs while high APE1 level confers resistance (Figure 2E‐G). To test this hypothesis, we determine the cytotoxicity of EGFR‐TKIs using a CCK8 assay after exogenous manipulation of APE1 expression. APE1 was successfully knocked down in HCC827/IR and PC‐9/ER cell lines via siRNA transfection and overexpressed in HCC827 and PC‐9 via lentiviral particles, both confirmed by Western blot. The gefitinib IC_50_ in APE1 overexpressing HCC827 and PC‐9 cells is increased compared to control lentiviral particle‐infected cells demonstrating increased resistance to EGFR‐TKIs (*P* < 0.01) (Figure 3A,B). Meanwhile, APE1 knockdown in resistant cell lines significantly sensitizes the cells to EGFR‐TKIs (*P* < 0.01) (Figure 3C,D), which further confirms that APE1 plays an important role in regulating cellular response and sensitivity to EGFR‐TKI treatment.

### 3.3 APE1 is associated with the EMT process in NSCLC cells

**Figure 4:**
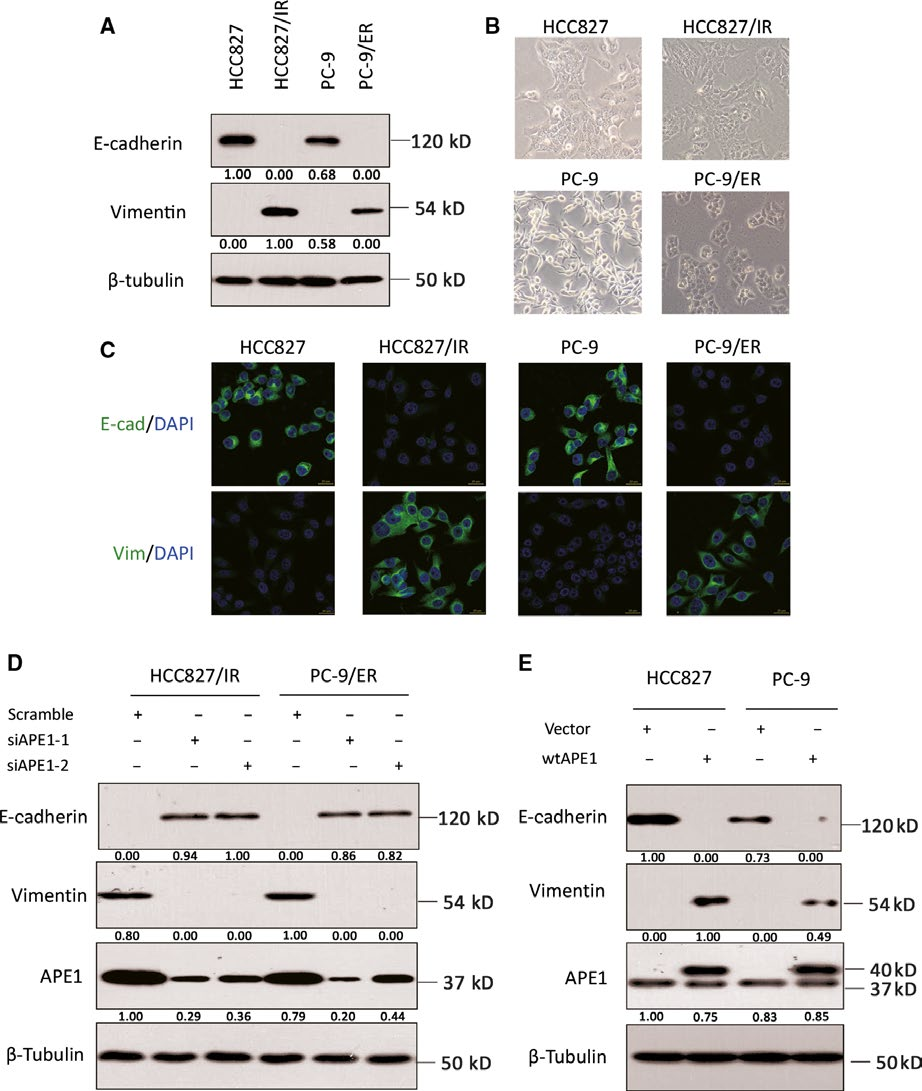
Manipulations of APE1 regulate the EMT process. The EMT markers in both cell lines with different APE1 status were measured by the epithelial marker E‐cadherin or the mesenchymal marker vimentin using Western blot (A), phase contrast microscopy (B), and immunofluorescent staining (C). Following knockdown of APE1 in HCC827/IR and PC‐9/ER cells via siRNA transfection, the expression of E‐cadherin, vimentin, and APE1 was determined by Western blot (D). Following overexpression of APE1 in EGFR‐TKI‐sensitive cells via lentiviral particles, relative expression levels of E‐cadherin, vimentin, and APE1 were determined by Western blot (E). Representative images or blots from at least three individual experimental repeats are shown in this figure

Epithelial‐to‐mesenchymal transition has been considered one of the most important mechanisms of tumor progression. Therefore, we confirm that EMT occurs in two EGFR‐TKI‐resistant cell lines by analyzing the epithelial marker E‐cadherin and the mesenchymal marker vimentin using Western blot assay (Figure 4A). To test if APE1 is involved in the EMT process during the development of acquired resistance to EGFR‐TKIs, EMT markers are checked in both the APE1 overexpressing EGFR‐TKI‐sensitive cells and APE1 knockdown EGFR‐TKI‐resistant cells. As shown in Figures 4E and S1B, overexpressing APE1 in EGFR‐TKI‐sensitive cells, where APE1 is minimally expressed, produces an EMT‐like phenotype including a transition in morphology involving E‐cadherin downregulation and vimentin upregulation. On the other hand, when APE1 expression is knocked down in EGFR‐TKI‐resistant cells where EMT has occurred, the epithelial phenotype is restored (Figures 4D and S1A). The EMT markers in NSCLC cells with varying APE1 expression status are further confirmed morphologically via phase contrast microscope and immunofluorescence (Figure 4B,C). The results demonstrate that a low‐expression level of APE1 is important in maintaining the epithelial phenotype in EGFR‐TKI‐sensitive cells, while elevated APE1 facilitates EMT during the development of acquired resistance to EGFR‐TKIs in NSCLC.

### 3.4 APE1 regulates EMT through TGF‐β signaling

**Figure 5:**
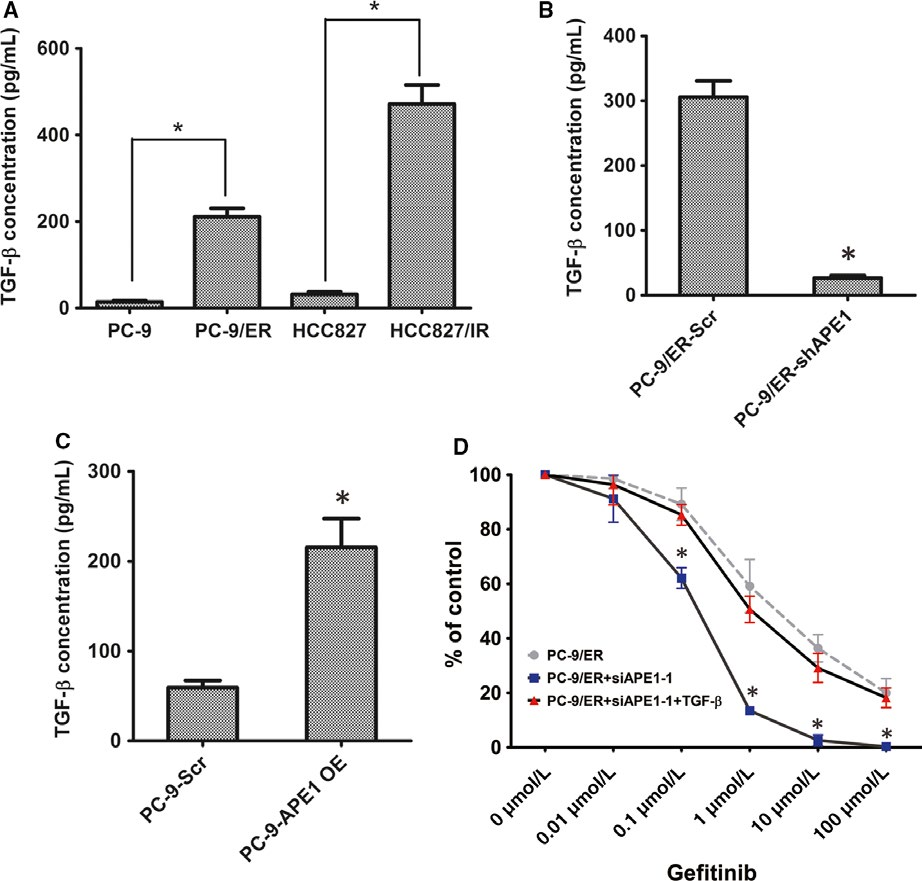
APE1 regulates EMT through TGF‐β signaling. TGF‐β secretion level was evaluated by ELISA in EGFR‐TKI‐resistant cell lines and their parental sensitive cells (A). The 24‐hour cell‐free supernatants of tissue culture from PC‐9 with or without APE1 overexpression and PC‐9/ER with or without APE1 knockdown were collected 48 h post‐transfection or infection and then evaluated by ELISA (B and C). Recombinant TGF‐β protein was added to the culture medium of APE1 knockdown PC‐9/ER cells, treated with increasing concentrations of gefitinib, and then, the cytotoxicity of EGFR‐TKI was determined by CCK8 assay (D). To exclude the impact of APE1 manipulation on cell growth, various gefitinib dose treatments of each group have been normalized to the readout of 0 μmol/L (DMSO only) treatment. Mean values of at least three individual experimental repeats are shown as the mean ± SD. * indicates a statistically significant difference when compared with the same treatment dose as its parental cell (*P *<* *0.01)

TGF‐β signaling plays a core regulatory role in the EMT process, and we previously demonstrated that APE1 promotes TGF‐β transcription in osteosarcoma, a malignancy derived from mesenchymal tissue.^9^ To further confirm this regulatory relationship in NSCLC epithelial cancer, TGF‐β secretion from cancer cells was evaluated by ELISA. We initially showed that TGF‐β secretion is significantly elevated in EGFR‐TKI‐resistant cell lines (Figure 5A). Conditioned medium was obtained from PC‐9 cells, with or without overexpression of APE1, and from PC‐9/ER with or without knockdown of APE1, upon culturing for 24‐hours in vitro and collected at 48 hours post‐transfection or infection. ELISA data shows that TGF‐β secretion is downregulated in APE1 knockdown cells compared to scrambled shRNA infected PC‐9/ER cells, whereas TGF‐β secretion is upregulated in APE1 overexpressing cells (Figure 5B,C). These results, in agreement with the EMT alteration pattern, strongly suggested that the influence of APE1 on EMT could be exerted through its control of TGF‐β in both epithelial and mesenchymal cancers. The rescue experiments were carried out by adding recombinant TGF‐β protein to the culture media of APE1 knockdown PC‐9/ER cells at a final concentration of 5 ng/mL. As a result of TGF‐β addition, APE1 knockdown PC‐9/ER cells regained resistance to gefitinib (*P* < 0.01), which was measured by CCK‐8 assay (Figure 5D). Taken together, our current data demonstrate that APE1 promotes EGFR‐TKI resistance in NSCLC cells by facilitating EMT, which is mediated by its control of TGF‐β expression.

### 3.5 The APE1 redox inhibitor E3330 suppresses EMT and restores the responsiveness to EGFR‐TKIs in vitro and in vivo

**Figure 6:**
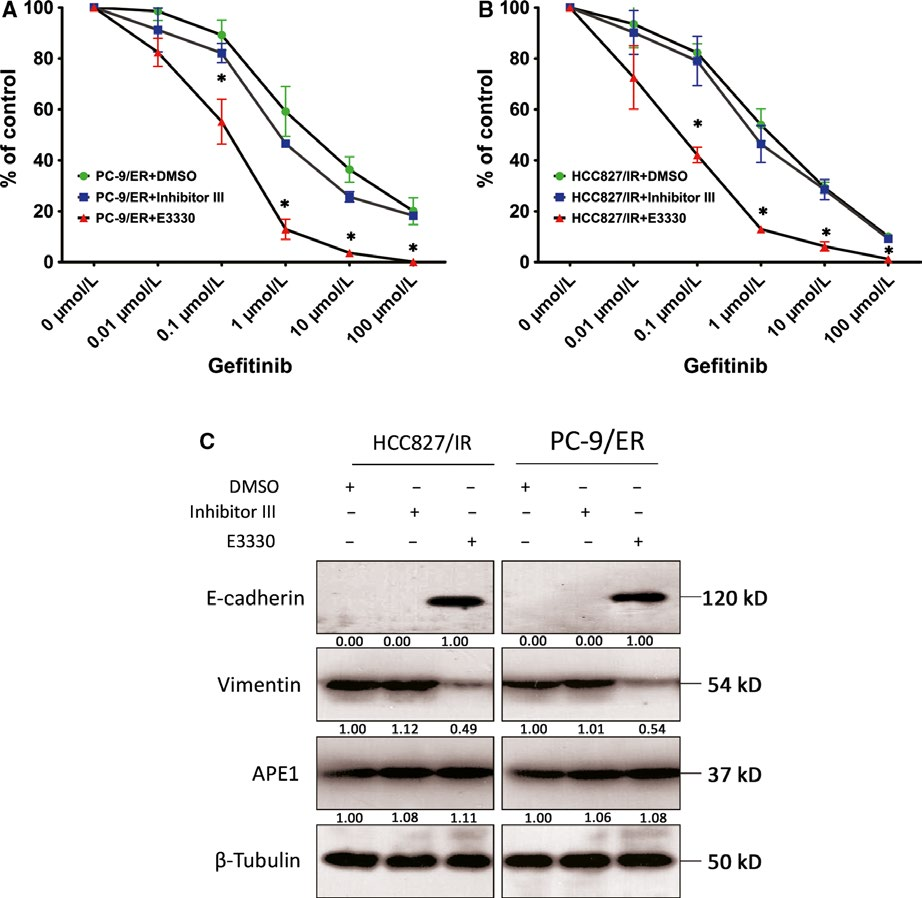
The redox activity of APE1 in EGFR‐TKI response and EMT. HCC827/IR and PC‐9/ER cells were treated with increasing concentrations of gefitinib and APE1 inhibitors, including 10 μmol/L inhibitor III (inhibits endonuclease activity) and 10 μmol/L E3330 (inhibits redox activity), with subsequent determination of the cytotoxicity of EGFR‐TKI by CCK8 assay (A and B). To exclude the impact of APE1 manipulation on cell growth, various gefitinib dose treatments of each group have been normalized to the readout of 0 μmol/L (DMSO only) treatment. The EMT markers were determined by Western blot in cells treated with each inhibitor at 10 μmol/L (C). The mean values of at least three individual experimental repeats are shown as the mean ± SD, * Statistically significant difference when compared with the DMSO group at the same gefitinib dose (*P *<* *0.05)

To evaluate if it is clinically feasible to utilize APE1 inhibitors to restore the responsiveness to EGFR‐TKIs in patients with acquired resistance, two activity‐specific inhibitors, including inhibitor III,^17^ which is specific for the endonuclease activity on abasic DNA, and E3330,^18^ which is specific for redox activity, were introduced. As shown in Figure 6A,B, E3330 significantly restores sensitivity to gefitinib in HCC827/IR and PC‐9/ER cells (*P* < 0.01) when compared to inhibitor III and vehicle (DMSO). As previously documented, treatment using these inhibitors had similar effects as compared to introducing individual functional mutants of APE1.^19^ These results suggest that APE1 redox activity, rather than DNA repair activity, is involved in EGFR‐TKI resistance and that this redox activity controls the EMT process. Then, we analyzed the EMT markers by Western blot in cells treated with either one or the other inhibitor and found that the epithelial phenotype was reversed in E3330‐treated cells but not in cells treated with inhibitor III (Figures 6C and S2). These data further confirm and are consistent with the premise that APE1 redox activity is the key functional component exerting a regulatory effect on EMT and subsequent EGFR‐TKI resistance.

**Figure 7:**
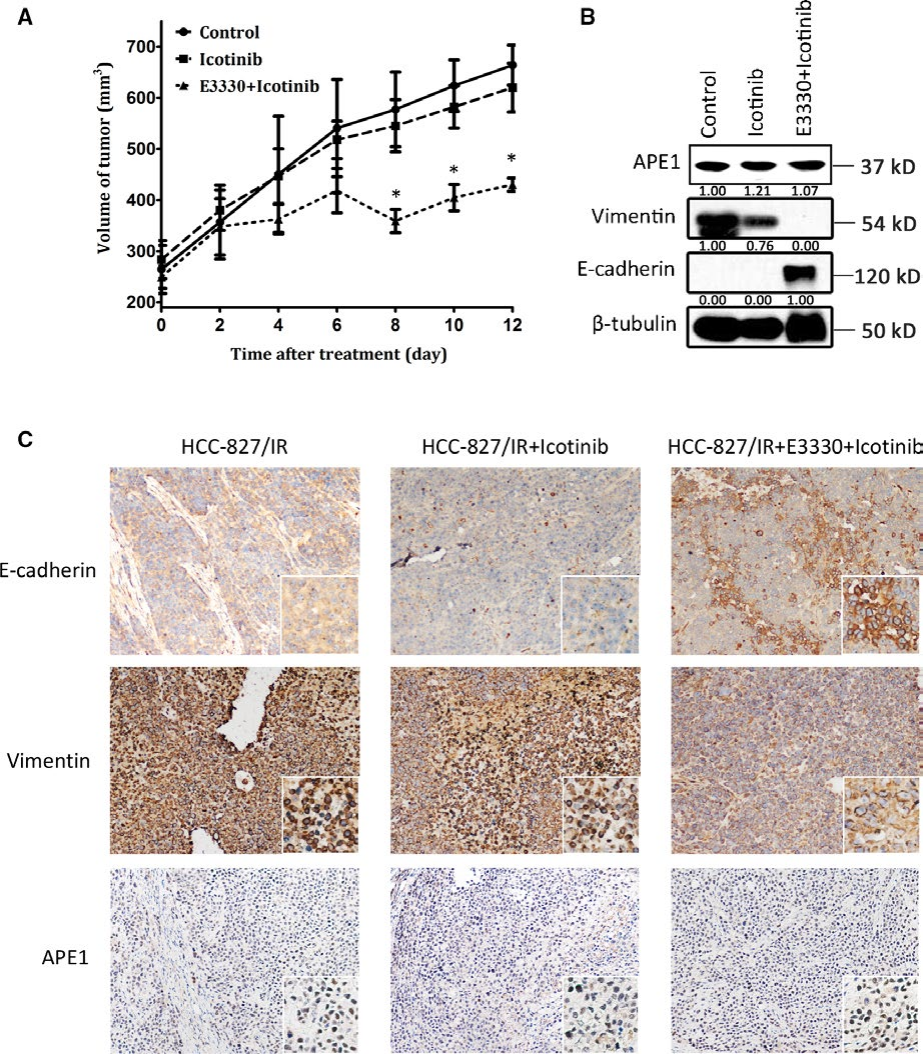
APE1 promotes the metastatic potential of xenograft lung cancer cells in nude mice. One million HCC827/IR cells were inoculated subcutaneously into nude mice which were then treated with icotinib at 0 mg/kg, 70 mg/kg alone, and 70 mg/kg together with E3330 at 25 mg/kg (n = 6 mice or 12 xenografts/group). Xenograft growth was measured in two dimensions, and volume was recorded (tumor size (mm^3^) = (maximum diameter ×minimum diameter^2^)/2) (A). After daily treatment for 12 d, the nude mice were sacrificed, and histologically intact xenografts were collected. The expression of APE1, E‐cadherin, and vimentin in the xenograft extracts was detected by Western blot (B). EMT analyses of tissue slides were performed via IHC staining of E‐cadherin and vimentin (C). The mean values of the 12 xenografts of each group are shown as the mean ± SD, * Statistically significant difference when compared with the control group (*P *<* *0.05)

We further investigated whether the in vitro restoration of EGFR‐TKI responsiveness by E3330 could be repeated in xenograft models in vivo. As an equivalent alternative to gefitinib, we utilized the first‐generation EGFR‐TKI, icotinib, which has been clinically proven to be equivalent to gefitinib.^1^, ^20^ HCC827/IR cells were injected into nude mice and treated when the xenograft volume reached approximately 250 mm^3^ with icotinib or icotinib combined with E3330 or vehicle alone for 12 days, as the nude mice cannot tolerate tumor load for a longer time. The growth of xenografts in the three groups was monitored and shown in Figure 7A. The result, in agreement with in vitro data, suggests that E3330, in combination with EGFR‐TKI, suppresses EGFR‐TKI‐resistant cancer cell growth (*P* < 0.01), whereas the tumor continues vigorously growing in the EGFR‐TKI alone treated group and vehicle control group. To confirm that the EMT process can be reversed in the E3330‐treated group in vivo, a Western blot assay on xenograft extracts (Figure 7B) and an IHC assay on tissue slides (Figure 7C) were performed to show the change of EMT biomarkers in all three groups. The data shown in Figure 7B suggests that E3330‐treatment upregulates the epithelial marker in HCC827/IR cells without interfering with APE1 expression level. We noted that icotinib treatment slightly downregulates vimentin expression without inducing E‐cadherin expression. IHC results agree with Western blot results showing that E3330 in combination with icotinib treatment significantly upregulates E‐cadherin expression while decreasing vimentin expression. Icotinib treatment alone has no significant effects on either EMT marker, suggesting that E3330 could, at least, partially reverses the EMT phenotype in HCC827/IR cells, which is in agreement with in vitro experiments. Considering APE1 expression is not affected by E3330/Icotinib combinational treatment, these results provide support for the hypothesis that the redox activity of APE1 is critical for EMT in EGFR‐TKI‐resistant NSCLC cells.

### 3.6 APE1 is elevated in T790M‐negative EGFR‐TKI‐resistant patients

**Figure 8:**
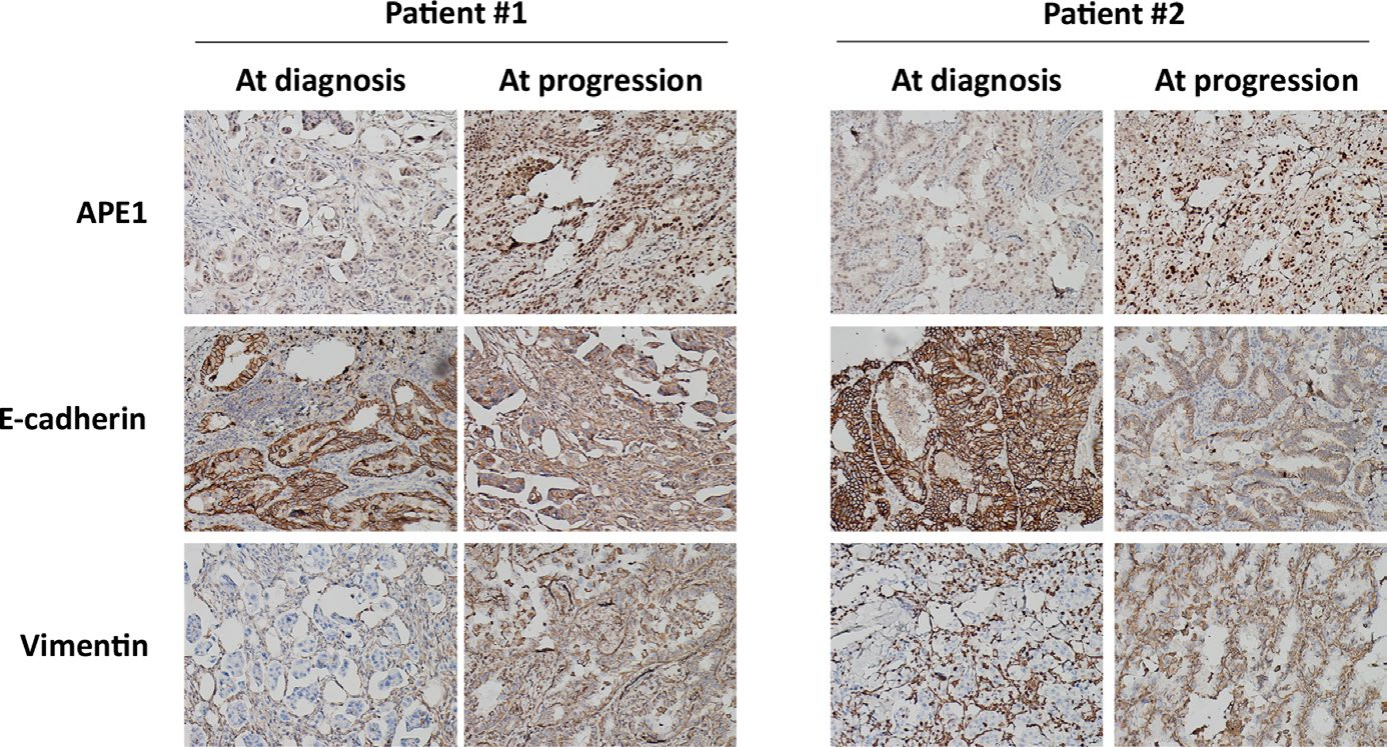
APE1 is elevated in T790M‐negative EGFR‐TKI‐resistant patients. APE1 and the EMT biomarkers E‐cadherin and vimentin were assayed by IHC in the tumors of two patients receiving rebiopsy after progression following treatment with first‐generation EGFR‐TKI, and these markers were compared with pretreatment biopsy tissue. Both patients underwent rebiopsy at disease progression with core needle, and the tumor was determined to be T790M negative by the Cobas method

To test the proposed mechanisms determined from in vitro cell models, we compared the APE1 protein levels in pre‐ and post‐treatment biopsy tissue of two patients receiving rebiopsy after progression and following treatment with first‐generation EGFR‐TKIs. Tumor tissue from Patient #1 was initially characterized with an L858R mutation when diagnosed with left lung adenocarcinoma, then gefitinib was given at 250 mg, po., qd. After 10.8 months following the first gefitinib dose, disease progression in the left lung was confirmed by CT scan. Tumor tissue from Patient #2 was initially tested and characterized as an exon 19 deletion when diagnosed with left lung adenocarcinoma. Erlotinib was then given at 150 mg, po., qd. After 20.3 months following the first erlotinib dose, disease progression to the brain was confirmed by an MRI scan. Both patients underwent rebiopsy by core needle at disease progression and both tumors were determined to be T790M negative by the Cobas method. APE1 and EMT biomarkers, including E‐cadherin and vimentin were tested using IHC assay. As shown in Figure 8, APE1 was elevated in the rebiopsy tissue of both patients compared with the pretreatment tissue. In agreement with APE1 level, vimentin was upregulated in the rebiopsy tissue whereas E‐cadherin was downregulated. This pilot clinical study further confirmed our in vitro results, strongly suggesting that APE1 could be an emerging key player in EGFR‐TKI responsiveness through regulating EMT.

## 4 DISCUSSION

While EGFR‐TKIs significantly improve the survival and quality of life in advanced NSCLC patients carrying EGFR mutations, acquired resistance to these inhibitors limits their clinical benefit, thus creating challenges for treatment and management. In the current study, we describe a novel and pivotal role for APE1 in EGFR‐TKI resistance, which suggests that APE1 expression could predict the therapeutic effect of EGFR‐TKI treatment. We further demonstrated that, through its redox regulation of TGF‐β, APE1 plays a role in the control of EMT and mediation of EGFR‐TKI resistance. The APE1 redox inhibitor E3330 restored sensitivity to EGFR‐TKIs in established resistant cell lines, further implying efficacy for therapeutic combinations of specific APE1 inhibitors and EGFR‐TKIs with the potential of restoring their clinical benefit.

The sensitizing effects of APE1 on other conventional anticancer agents, including chemotherapeutic drugs and irradiation, have been observed previously.^21^, ^22^, ^23^ Both chemotherapeutic agents, such as MMS, and irradiation, such as X‐rays, generate numerous DNA lesions that, if unrepaired, cause cell death. Thus, the mechanistic explanations of previous reports regarding the sensitizing effects of APE1 on anticancer agents were largely correlated with its essential activity in DNA repair. However, EGFR‐TKIs, as the first type of targeted drugs in NSCLC, are specifically targeted to driver mutations of EGFR and inhibit their prosurvival activity, an effect which lacks a direct link to DNA damage and repair. As seen in our experiments, a potent DNA repair activity inhibitor of APE1, inhibitor III, failed to restore the sensitivity to EGFR‐TKI, further supporting our hypothesis. Our intriguing and novel observation links, for the first time, the redox activity of APE1 with acquired resistance to EGFR‐TKIs, which has potentially important clinical significance. Despite the controversies, many laboratories are currently working on the development of redox inhibitors of APE1, including a derivate of E3330, APX3330 which is now in phase II clinical trials for pancreatic cancer and hematopoietic malignancies (via personal communication). With respect to these ongoing trials, the clinical use of APE1 redox inhibitors is not only reasonable but also feasible in the near future to reverse, or at least attenuate, resistance to the EGFR‐TKIs.

Apart from the impact of the initial response to DNA damaging agents, APE1 levels have no impact on the initial response to EGFR‐TKIs.^24^ However, elevated APE1 levels in pretreatment tissue showed a significant inverse correlation with the duration of the response; in other words, the speed of acquired resistance development increases with elevated APE1 levels. We consider this pattern to be related to the onset of EMT initiation where APE1 plays a pivotal role. We recently discovered that APE1 protein is also present in human serum and, more importantly, is elevated in NSCLC patients, and correlates with platinum‐containing chemotherapy responses. When tracing serum APE1 throughout treatment, APE1 levels change during chemotherapy and reflect therapeutic outcomes.^25^ Based on this observation, we initiated a trial to monitor changes in serum APE1 levels during EGFR‐TKI treatment. If a correlation between serum APE1 level and EGFR‐TKI response can be observed prior to the actual imaging progression, we could predict resistance events and prophylactically add APE1 inhibitors or other possible interventions by real‐time monitoring.

To the best of our knowledge, this is the first study confirming that APE1 exerts a regulatory function in EMT. High APE1 expression in tumor tissue has been associated with advanced stage, poorer prognosis, metastasis, and drug resistance according to clinical data.^26^ Several models have been proposed accounting for APE1′s functions in promoting the malignant phenotype of different cancers. For the first time, APE1 has been associated with an important cellular process, EMT, which could account for the progression, particularly the metastasis and drug resistance of certain epithelial cancers.^27^ The newly established correlation between APE1 and EMT provides a plausible explanation for the malignant phenotypes associated with overexpression of APE1. With respect to the possible relation between EMT and cancer stem cells (CSCs), APE1 might also play a role in CSC maintenance, which has been supported by some circumstantial evidence.^28^ The causal functional link existing between APE1 and CSC, as well as its relationship with the EMT, should be further investigated considering that the alterations of these stem cells are responsible for EGFR‐TKI resistance.^29^ To further substantiate the important role of APE1 in EMT, we discovered that E3330 can effectively suppress the EMT process and the peritoneal and liver metastasis of cervical cancer cells in nude mice which suggests that APE1 plays a pivotal role in tumor metastasis through promoting EMT (unpublished data).

Our current data provide evidence that APE1 regulates TGF‐β secretion and that increased APE1 is associated with increased TGF‐β secretion, which initiates EMT and impedes cellular response to EGFR‐TKIs. It is noteworthy that Sakai et al^30^ reported previously that TGF‐β mRNA was elevated in APE1 knockdown lung carcinoma cell line A549 and cervical cancer cell line HeLa. They also linked the downregulation of APE1 to increased cellular motility. However, clinical studies have shown that higher APE1 expression is associated with more advanced stages and poorer prognosis of cancer. This single report contradicted previous clinical evidence that overexpression of APE1 is correlated with malignancy of cancer and thus should be further evaluated. As a very complex process, the molecules and mechanisms involved in regulating EMT are not yet fully identified. Transcription factors including Snail, Slug, and ZEB1/2 have been shown to be important for reprogramming of the gene expression profile during EMT. One might wonder whether other transcription factors previously reported to be associated with APE1 redox activity, such as STAT3 and HIF‐1, might participate in the process of EMT reversion induced by APE1 inhibition.^31^, ^32^ We tested both STAT3 and HIF‐1 DNA binding activities in EGFR‐TKI‐resistant cell lines and their parental lines via electrophoretic mobility shift assay (EMSA) and found that the DNA binding activity of both TFs retains similar levels in both EGFR‐TKI‐resistant and EGFR‐TKI‐sensitive cell lines. We did confirm that APE1 manipulation by shRNA or overexpression vectors can affect the DNA binding activities of both TFs (Figure S3). These data suggest that APE1 expression regulates the activities of both TFs but they probably have no significant roles in EGFR‐TKI resistance in our current setting. Considering the transcriptional regulatory role of APE1, it is possible that APE1 may also directly regulate other key transcription factors that promote EMT. Additional work in our laboratory is underway to investigate this possibility.

Taken together, the current study reveals a significant role of APE1 in EGFR‐TKI resistance via novel regulatory effects on EMT in NSCLC and provides evidence supporting the involvement of APE1 in a malignant progression through EMT. More importantly, this study suggests the possible predictive role of APE1 levels in tissue or serum for EGFR‐TKI responsiveness and sheds light on the future clinical utility of APE1 redox inhibitors to overcome EGFR‐TKI resistance.

## CONFLICT OF INTEREST

None declared.
