# Supplementary figures and images for: The regulatory role of APE1 in epithelial‐to‐mesenchymal transition and in determining EGFR‐TKI responsiveness in non‐small‐cell lung cancer
Source: Cancer Med. 2018 Aug 14;7(9):4406–19. doi: 10.1002/cam4.1717 (PMC6144255; doi:10.1002/cam4.1717)

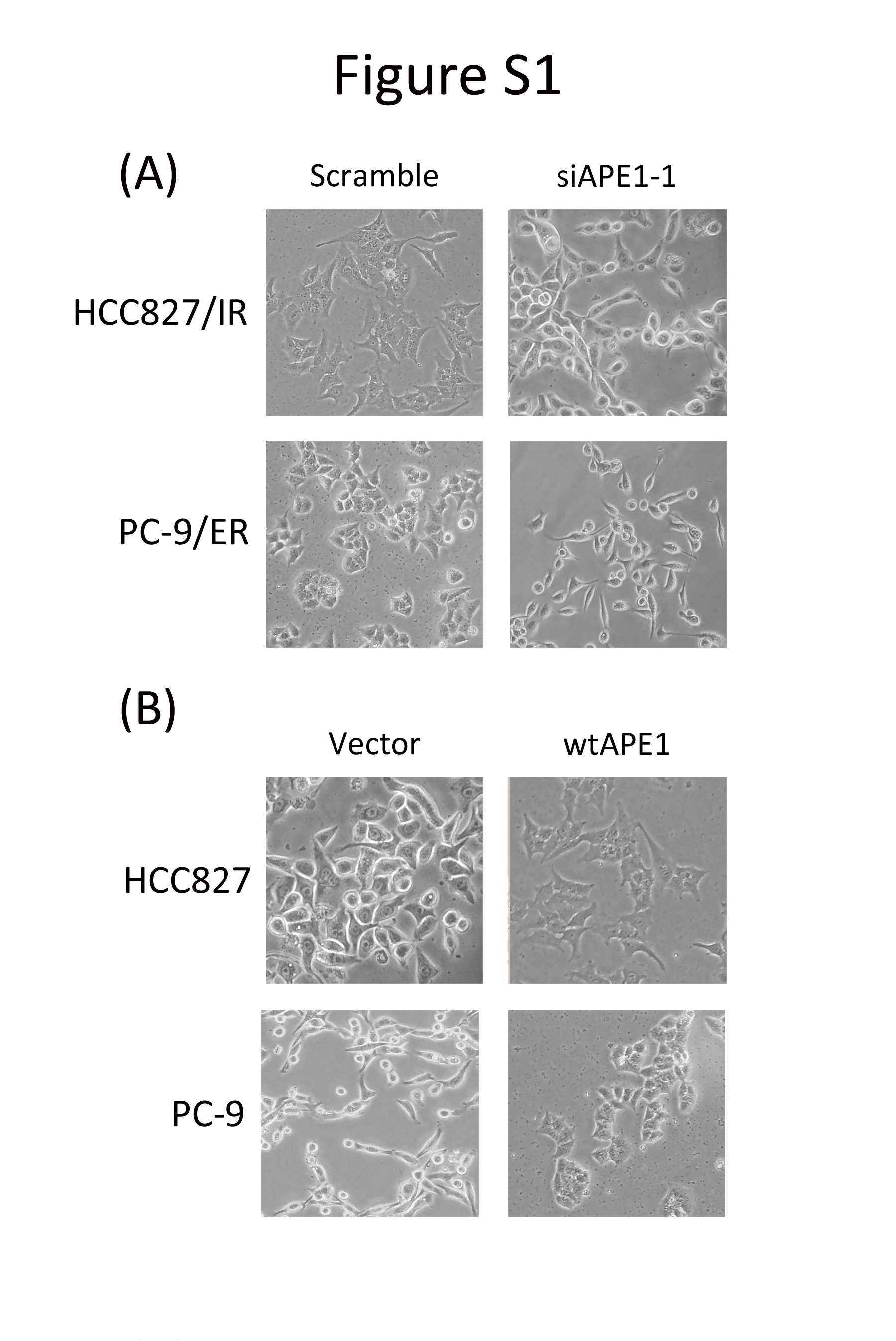

Supplement: Supplementary file 1 [file CAM4-7-4406-s001.tif]

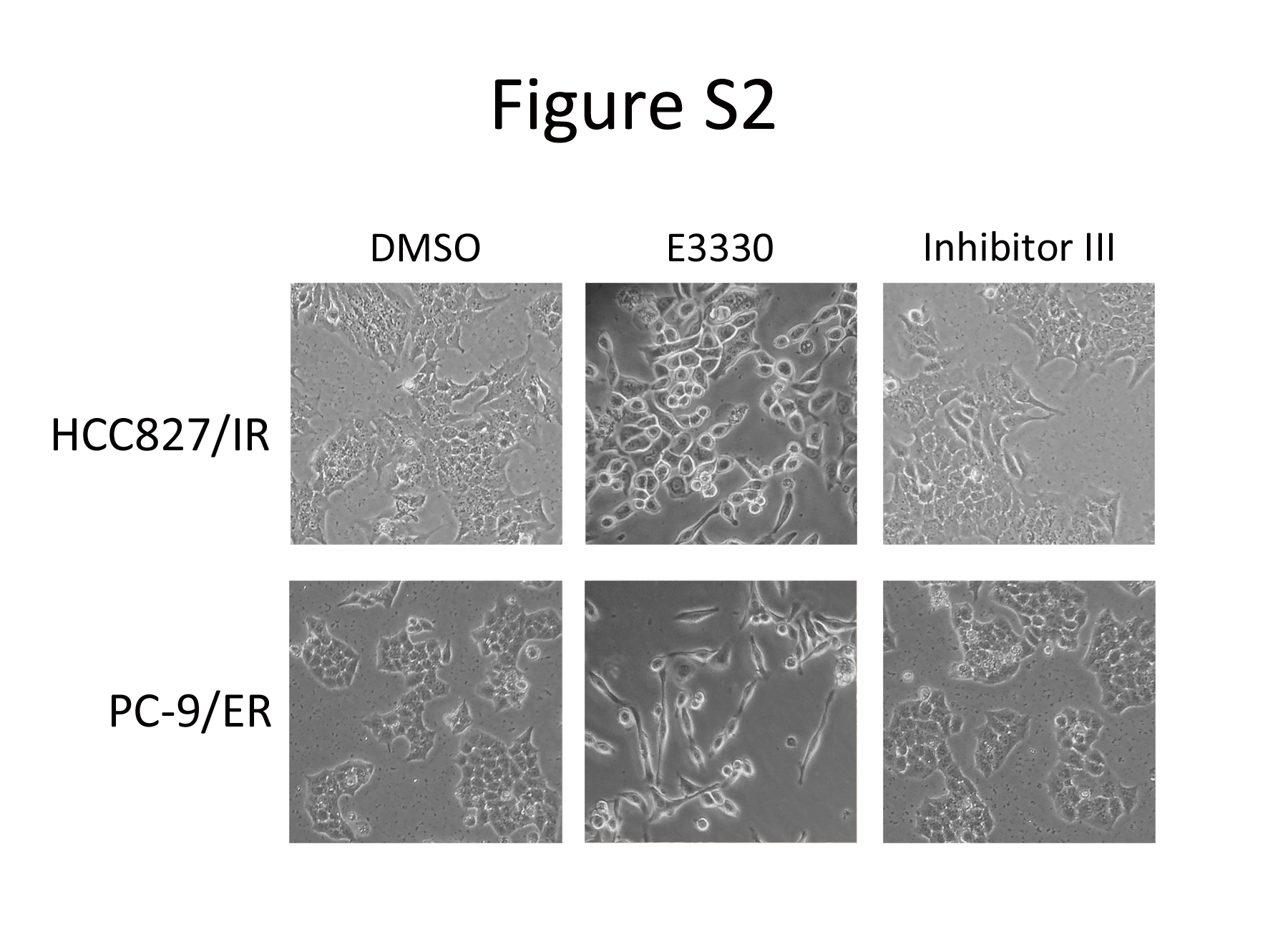

Supplement: Supplementary file 2 [file CAM4-7-4406-s002.tif]

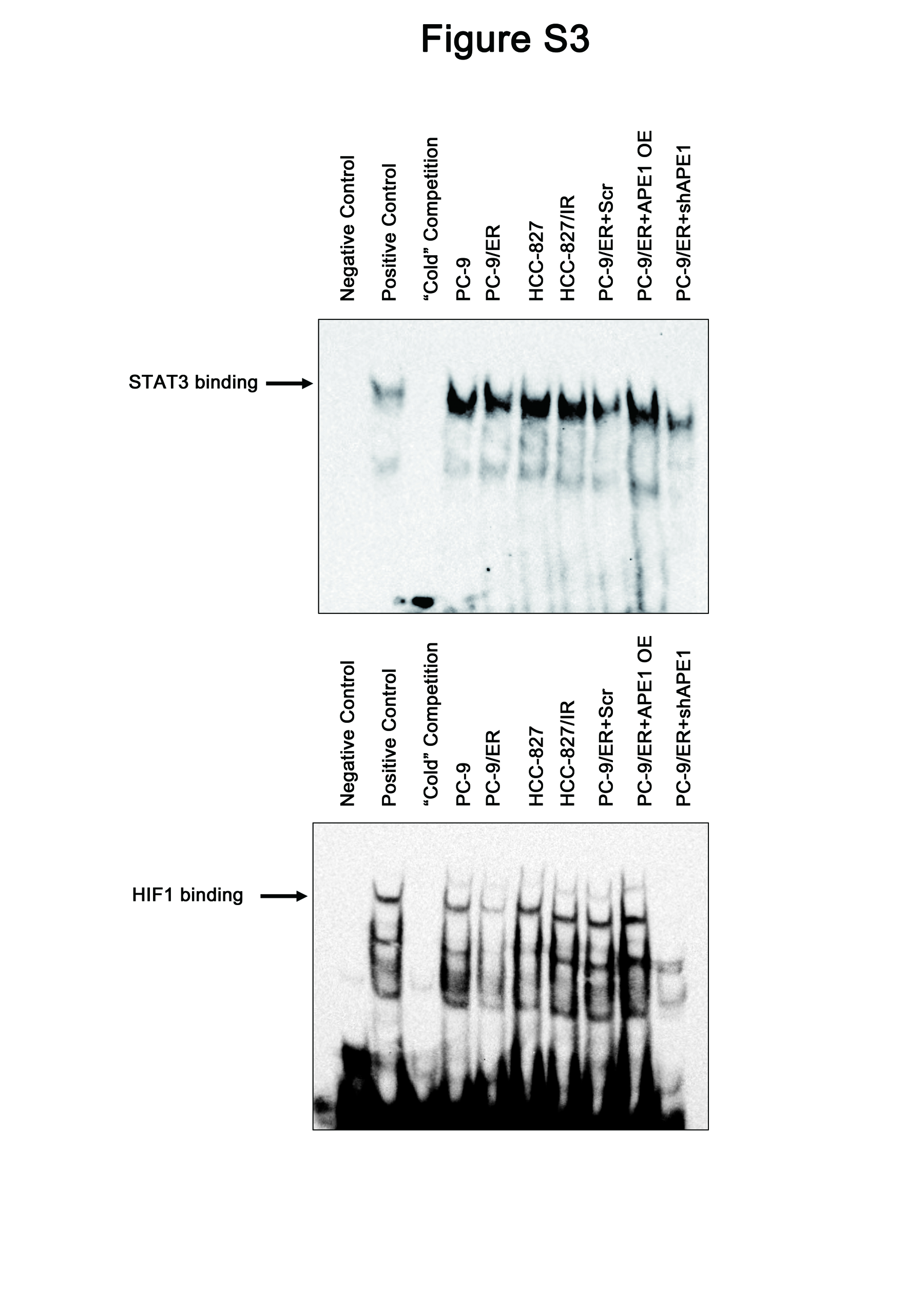

Supplement: Supplementary file 3 [file CAM4-7-4406-s003.tif]
